# Wildfire plumes in the Western US are reaching greater heights and injecting more aerosols aloft as wildfire activity intensifies

**DOI:** 10.1038/s41598-022-16607-3

**Published:** 2022-07-20

**Authors:** Taylor Y. Wilmot, Derek V. Mallia, A. Gannet Hallar, John C. Lin

**Affiliations:** Department of Atmospheric Sciences, University of Utah, Salt Lake City, UT USA

**Keywords:** Atmospheric science, Climate change, Environmental impact

## Abstract

By producing a first-of-its-kind, decadal-scale wildfire plume rise climatology in the Western U.S. and Canada, we identify trends toward enhanced plume top heights, aerosol loading aloft, and near-surface smoke injection throughout the American West. Positive and significant plume trends suggest a growing impact of Western US wildfires on air quality at the local to continental scales and support the notion that wildfires may have an increasing impact on regional climate. Overlap of identified trends with regions of increasing wildfire emissions and burn severity suggests a link to climate driven trends toward enhanced wildfire activity. Further, time series of plume activity point to a possible acceleration of trends over recent years, such that the future impacts to air quality and regional climate may exceed those suggested by a linear fit to the multi-decadal data. These findings have significant implications for human health and exacerbate concern for the climate–wildfire connection.

## Introduction

Aerosols emitted by wildfires^1^ affect both air quality and climate. Aerosol particulate matter with an aerodynamic diameter less than 2.5-microns (PM_2.5_) has been linked to enhanced population level mortality^2,3^ and increased instances of respiratory illness^4–7^. Relative to PM_2.5_ from non-fire sources (e.g., industrial emissions), mounting evidence suggests that wildfire emitted PM_2.5_ may be more toxic^5,8^, therefore posing a greater risk to human health. In terms of climate, aerosol loading from wildfires has a direct radiative impact in the form of absorption and scattering of incoming solar radiation by black carbon^9,10^ and organic aerosols^11^, respectively. Additionally, wildfire emitted aerosols generate indirect radiative effects by altering cloud microphysics^12,13^.

Over recent decades, Western US wildfire activity has become increasingly intense, with many mountainous regions demonstrating increases in annual fire area burned^14–16^, annual fire area burned at high-severity^14^, the number of large wildfires^16^, wildfire emissions of aerosol^17^, and in some cases high-altitude burning^18^. Similarly, portions of British Columbia demonstrate increases in wildfire emissions^17^. This shift in wildfire activity has been, in part, attributed to a changing climate, with strong links drawn to increasing temperatures and aridity^14,18–21^, reduced summertime precipitation^22^, and a declining snowpack^23,24^. Significant increases in extreme fire weather across the Western US over the years 1979–2020 have been attributed to increases in the vapor pressure deficit, decreases in relative humidity, and the dependence of these aforementioned shifts on increasing temperatures^21^. Wildfire activity in the Klamath Mountains/North Coast and Sierra Nevada regions of California, which are large contributors to wildfire activity at the scale of the Western US, has been linked to warming temperatures and increased atmospheric aridity^20^. Similarly, increases in high altitude burning across the Sierra Nevada, Middle Rockies, and Southern Rockies have also been attributed to drier conditions and warmer temperatures^18^. Global climate models indicate 2 °C of warming by the year 2050 would continue to exacerbate wildfire activity^25^, thereby increasing the air quality and climate impacts of large wildfires. Given the legacy of fire suppression in Western US forests, it is reasonable to expect these impacts to be further amplified^26^.

Wildfire plume rise, a result of buoyancy produced by combustion-derived heating, determines the altitude at which wildfire emitted aerosols are injected into the atmosphere, modulating the air quality and climate impacts of a given wildfire. The plume injection height often dictates the transport pathway of wildfire smoke. For smoke injections of equal mass, penetrative injections above the planetary boundary layer (PBL) enhance the potential for air quality impacts at regional to continental distances from the fire due to the efficiency of long-range transport and the potential for subsequent boundary layer entrainment^27–29^. Conversely, injections confined to the PBL pose a greater risk to local air quality as they are less vertically diluted^30^. Similarly, aerosol injection aloft is associated with a greater potential for climate forcing by wildfire aerosols, given the longer residence times^31,32^, reduced attenuation of incoming solar radiation at altitude^9,29^, and temperature dependence of radiative emissions from the smoke plume^33^. Further, the radiative effects of aerosol-cloud interactions are sensitive to the injection altitude of black carbon^34^. In extreme cases, deep pyro-convection may inject aerosols directly into the stratosphere^35,36^, producing a stratospheric aerosol mass that is comparable to a moderate volcanic eruption^37^. Injection of wildfire emitted aerosols above the tropopause is linked to positive top-of-the-atmosphere radiative forcing at the hemispheric scale^38^, reductions in surface temperature^39,40^, reductions in stratospheric ozone^41^, and disruptions of atmospheric circulations in the lower stratosphere^42,43^.

The heights of wildfire smoke plumes are a complex function of the wildfire heat flux, fire geometry, and the ambient meteorological profile^44^. While a weak correlation exists between the fire intensity and plume top height^30,45^, atmospheric stability acts to limit the buoyant plume rise^30,46^ by inducing plume injection/smoke detrainment. As the plume rises, environmental eddies entrain cold air into the plume, diluting the vertical momentum and therefore limiting the plume top height^47^. The effects of dynamic entrainment, produced by a strong horizontal wind^47^, result in a bent-over plume structure^48^. Furthermore, latent heat release offers a means to fuel additional plume rise, highlighting the importance of moist layers and moist convection to plume top height^30^. As testament to the role of moist convection, the inverted-V sounding, in which a moist layer sits atop a relatively deep and dry surface layer, has been positively linked to pyrocumulonimbus (deep pyro-convection; pyroCb) development and instances of stratospheric aerosol injection^33^.

Despite remaining uncertainties as to the exact governing equations of the wildfire plume rise and its expression in a modeling framework^44^, climate driven trends toward enhanced wildfire activity^14–24^ and related degradation of Western US air quality^17,49,50^ highlight the need for investigation into the evolving state of wildfire aerosol injection and the associated vertical mass distribution. At present, the temporal and spatial limitations of satellite sampling by space-borne sensors—i.e., the Multi-angle Imaging SpectroRadiaometer (MISR) and Cloud-Aerosol Lidar with Orthogonal Polarization (CALIOP)—prohibit a purely observational investigation of this topic. Plume top heights observed by MISR, which is limited to making observations during the late morning, indicate that the fraction of plumes penetrating above the PBL is highly sensitive to the definition of plume top. When the plume injection height is defined as the median MISR retrieved height for all pixels in a given plume, a mere 4–18% of North American plumes penetrate above the PBL^30,51,52^. However, when the plume injection height is defined as the maximum retrieved height for an individual plume, 48% of North American plumes are found to exceed the PBL^45^. Further, plume penetration above the PBL is complicated by the presence of diurnal cycles in fire intensity and lower atmospheric instability. When applied to global plume activity, the Freitas et al.^47,53^ plume rise model depicts a diurnal cycle in which ~ 25% of plumes penetrate above the PBL during morning hours, while the late afternoon maximum in fire intensity^54,55^ and enhanced lower atmospheric instability correspond to a value of ~ 53%^56^. Due to limited plume top observations and uncertainties surrounding plume rise modeling^44,45,57^, even less is known about the vertical distribution of detrained aerosol mass below the plume top. Observations from 20 controlled burns in the US Southeast suggest that the majority of plumes are either vertically well mixed or preferentially inject emissions aloft^58^. Coupled atmospheric—fire spread modelling, 3 -dimensional plume rise modeling, and detrained particle trajectory modeling suggest a tendency for plumes to deliver a majority of their aerosol mass toward the plume top^47,59,60^, while existing parameterizations for chemical transport modeling range from parabolic and Gaussian distributions to a constant dilution throughout the depth of the PBL^44,47,57^.

Given limitations in previous observational and modeling approaches, investigation into the evolving vertical distribution of wildfire injected aerosol mass requires a plume rise modeling framework that simulates both the plume top height and the vertical mass distribution in a manner supported by the limited observations and relevant theory. Application of such a framework to simulation of one-dimensional wildfire plumes at the regional-decadal scale may provide the sample size necessary for meaningful statistical analyses in the context of model uncertainty. Here we seek to generate a physics-based wildfire plume rise climatology that (i) leverages satellite burned area data, wildfire emissions, and atmospheric modeling outputs to provide regional scale understanding of plume activity beyond the limited satellite sampling, (ii) combines information on plume entrainment and mass conservation to estimate the vertical smoke detrainment profile of individual plumes, and (iii) when linked to wildfire PM_2.5_ emissions, allows the first estimate of how the vertical distribution of wildfire aerosol may evolve under climate driven trends toward increasingly intense wildfire activity.

## Results

**Figure 1:**
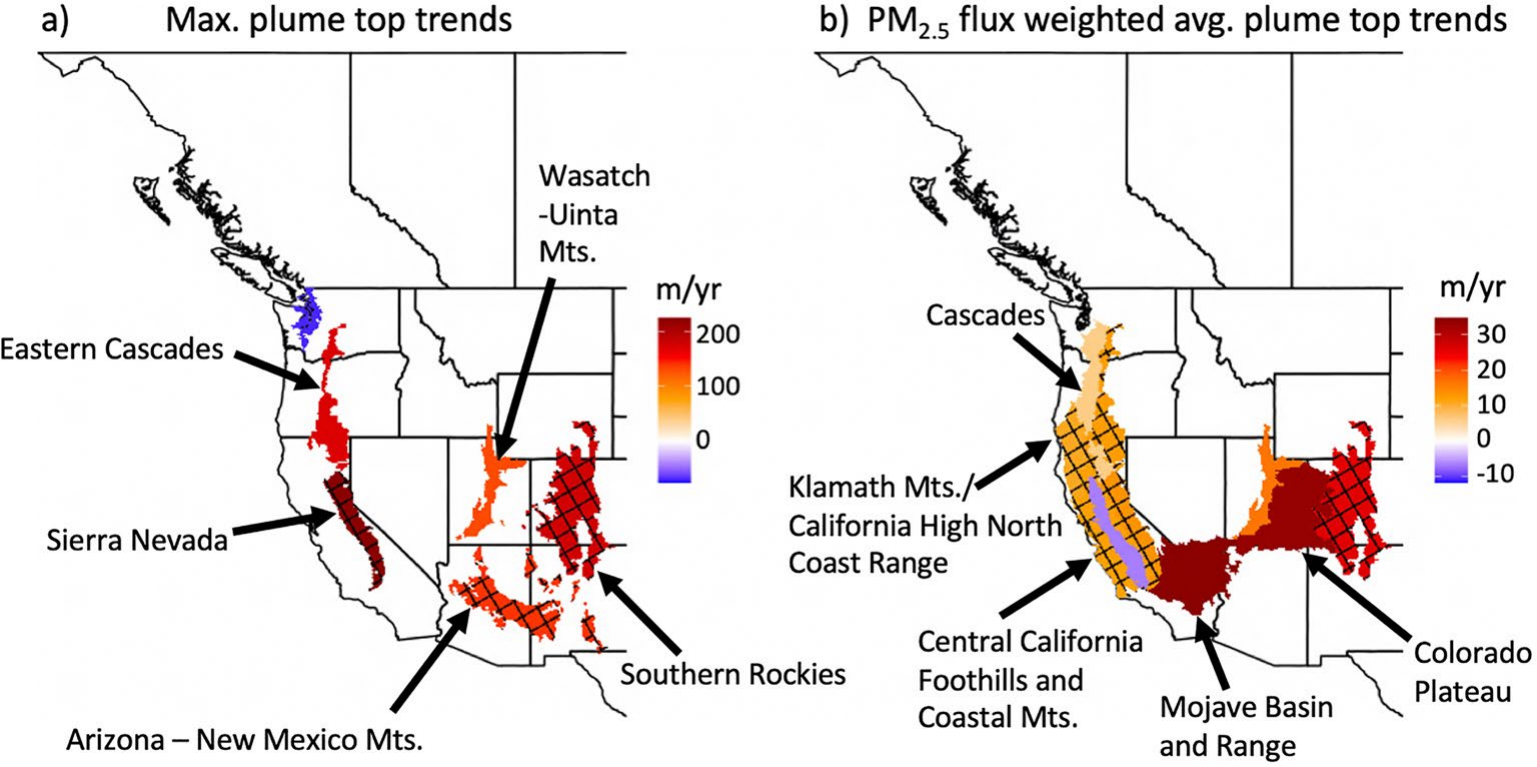
(**a**) Results for linear regressions trend analyses (2003–2020) of the maximum August–September plume top height for Western US ecoregions and Canadian ecoprovinces based on ~ 4.6 million plumes. (**b**) Same as (**a**), but for the PM_2.5_ flux-weighted average plume top height. Only trends significant at p < 0.1 are displayed, with a crosshatch overlaid on trends with p < 0.05. Ecoregions relevant to the results presented within this manuscript are labelled. Given the similarity of trends presented in (**b**) for the Sierra Nevada, Central California Foothills and Coastal Mountains, and Klamath Mountains/California High North Coast Range ecoregions, a visual distinction may be made by viewing Fig. 2. Complete mappings of United States level 3 ecoregions^66^ and Canadian ecoprovinces^67^ are available from the United States Environmental Protection Agency and the Government of Canada.

We use the Freitas et al.^47,53^ plume rise model (hereafter abbreviated as “F2010”, see “Freitas et al. plume rise model” section in “Methods” section), and an entrainment–mass conservation approach (see “Entrainment–mass conservation” section in “Methods” section) to estimate the vertical mass distribution of detrained smoke. Thus, we construct a plume rise climatology of ~ 4.6 million plumes occurring within the Western US and Western Canada during the months of August and September for the years 2003–2020. Model outputs are combined with PM_2.5_ emissions from the Quick Fire Emissions Dataset (QFED)^61^ and are considered in the context of PBL heights adopted from the Stochastic Time-Inverted Lagrangian Transport model (STILT; see “PBL estimates from STILT” section in “Methods” section)^62,63^. Linear regression and hinge fitting analyses (two-piece linear regressions, see “Trend analyses” section in “Methods” section) are used for insight into the trend of wildfire plume top heights and the vertical distribution of wildfire injected aerosol mass at the ecoregion scale. Ecoregions featuring prominently within the results are labeled in Fig. 1, with their corresponding number of simulated plumes per year presented in Table S1. Model evaluation is performed via comparison to the MISR plume heights dataset^64^. Emphasis is placed on August and September plumes given previously-identified wildfire emissions trends^17^ and the computational/storage expense of generating regional-decadal scale model inputs.

**Figure 2:**
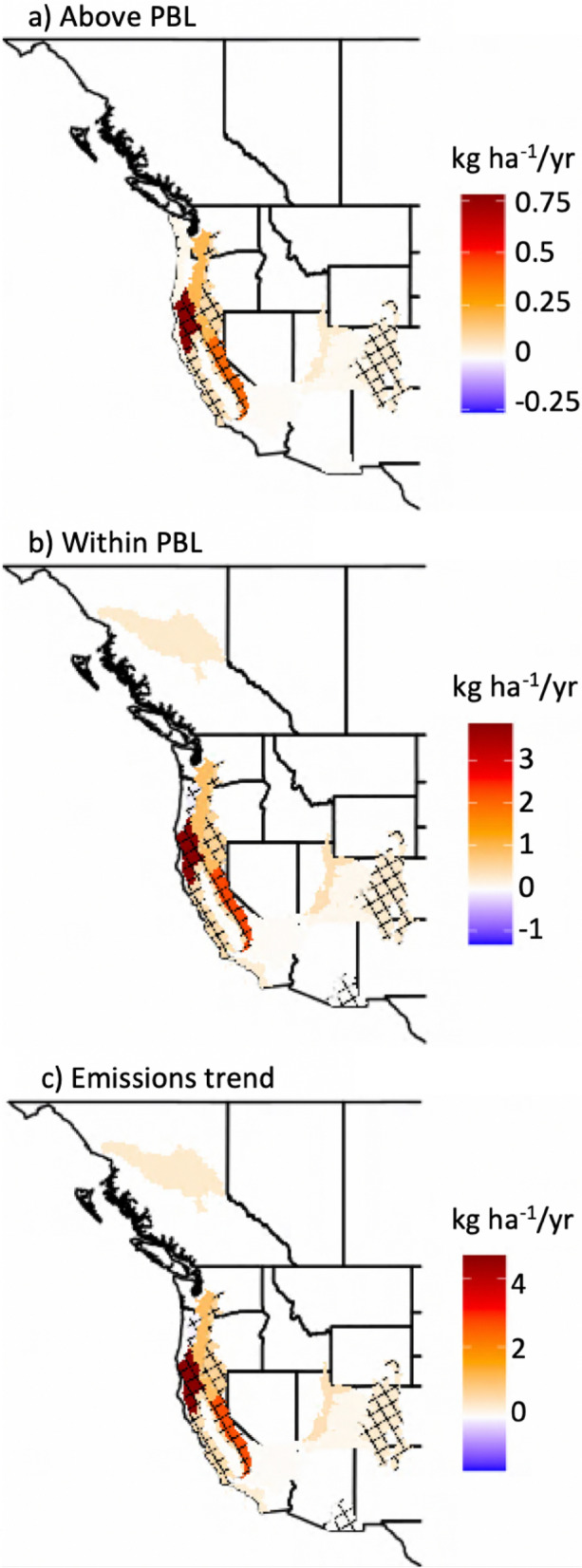
Results for trend analyses of the PM_2.5_ mass (**a**) injected above the PBL, (**b**) injected within the PBL, and (**c**) emitted by wildfires for Western US ecoregions and Canadian ecoprovinces during August and September of 2003–2020 based on ~ 4.6 million simulated plume rises. Only trends significant at p < 0.1 are displayed, with a crosshatch overlaid on trends with p < 0.05. Note the differences in the ranges represented by the color bars in (**a–c**).

Results of trend analyses should be considered within the context of potentially large uncertainties. Model inputs characterizing the wildfire heat flux and active burn area are influenced by satellite spatial resolution, the ability of clouds to obscure satellite observations, the assumption of a constant heat release per unit biomass burned, and the imperfect synthesis of burned area and heat release datasets (“Wildfire inputs” section). Further, though the model framework captures the core of the MISR observed plume top height distribution, there is evidence of a systemic underestimation of the frequency of the tallest plume tops, likely impacting the presented trend analyses.

### Trends in plume top height and aerosol mass injection

Ecoregion scale analyses within the Western US and Canada indicate that wildfire plume top heights are increasing throughout much of the mountainous Western US between the years of 2003 and 2020. In terms of the maximum plume top height during the months of August and September, we find trends as large as ~ 230 m per year in the Sierra Nevada ecoregion and trends in excess of 100 m per year in the Southern Rockies, Eastern Cascades, Arizona—New Mexico Mountains, and the Wasatch—Uinta ecoregions (Fig. 1a). If performed for the 5, 10, 30, or 100 largest plume top heights per year in each ecoregion, robust trends toward enhanced plume top heights persist in the Sierra Nevada and Eastern Cascades. Trend analyses considering the PM_2.5_ flux (kg PM_2.5_ emitted within the plume per second)—weighted average plume top height (Fig. 1b) further support the growth of plume top heights for the strongest emitting fires across much of the Western US, including notable trends in the mountain adjacent Colorado Plateau and Mojave Basin and Range ecoregions (> 30 m/year). While statistically insignificant, hinge fits (see “Trend analyses” section in “Methods” section) to the data suggest an acceleration of flux-weighted average plume top trends from ~ 2018 to 2020, highlighting the unprecedented nature of recent Western US wildfire activity relative to 2003—mid-2010s. In the Klamath Mountains, Sierra Nevada, and Southern Rockies, we find hinge fit trends (2017/2018–2020) of 99.0, 201.2, and 256.1 m/year, respectively, representing enhancements by a factor of 9–15 relative to results from linear regression (2003–2020). The presence of positive, significant (p < 0.1), and potentially accelerating trends throughout much of the mountainous Pacific Northwest and California, as well as the Southern Rockies, overlaps with previously identified potential wildfire emissions “hotspots”^17^.

We further undertake an examination of trends in the most extreme fire activity, as represented by pyrocumulonimbus (pyroCb) formation, defined by especially tall plumes with a plume top temperature <  − 38 °C (the homogenous freezing temperature for liquid water) being leveraged as a pyroCb proxy^65^. We find the possibility of increasingly frequent pyroCb activity within the Colorado Plateau (~ 0.12 pyroCb hours/year). Although trends for other ecoregions and the Western US/Canada as a whole are statistically insignificant, this climatology finds the first occurrence of pyroCb development in 6 ecoregions between 2017 and 2020, demonstrating an acceleration in recent years. These ecoregions include the Colorado Plateau, Southern Rockies, Sierra Nevada, Wasatch-Uinta, Mojave Basin and Range, and Northwestern Great Plains. Hinge fits further support the possibility of a recent uptick in pyroCb activity, with hinged trends (p > 0.1; 2018–2020) of 2.72, 2.15, and 0.43 pyroCb hours/year for the Colorado Plateau, Southern Rockies, and Sierra Nevada, respectively. A comparison of simulated pyroCb activity for 2013 to the observational inventory of pyroCb activity constructed by Peterson et al.^65^ indicates that this model framework reasonably captured the pyroCb formation above the Pony/Elk and Beaver Creek fires in Idaho, while failing to reflect the observed pyroCb activity above the Rim fire in California. Should pyroCb trends present greater clarity over the coming years, this result would support the growing concerns surrounding pyroCb induced warming of the stratosphere^38,39^.

**Figure 3:**
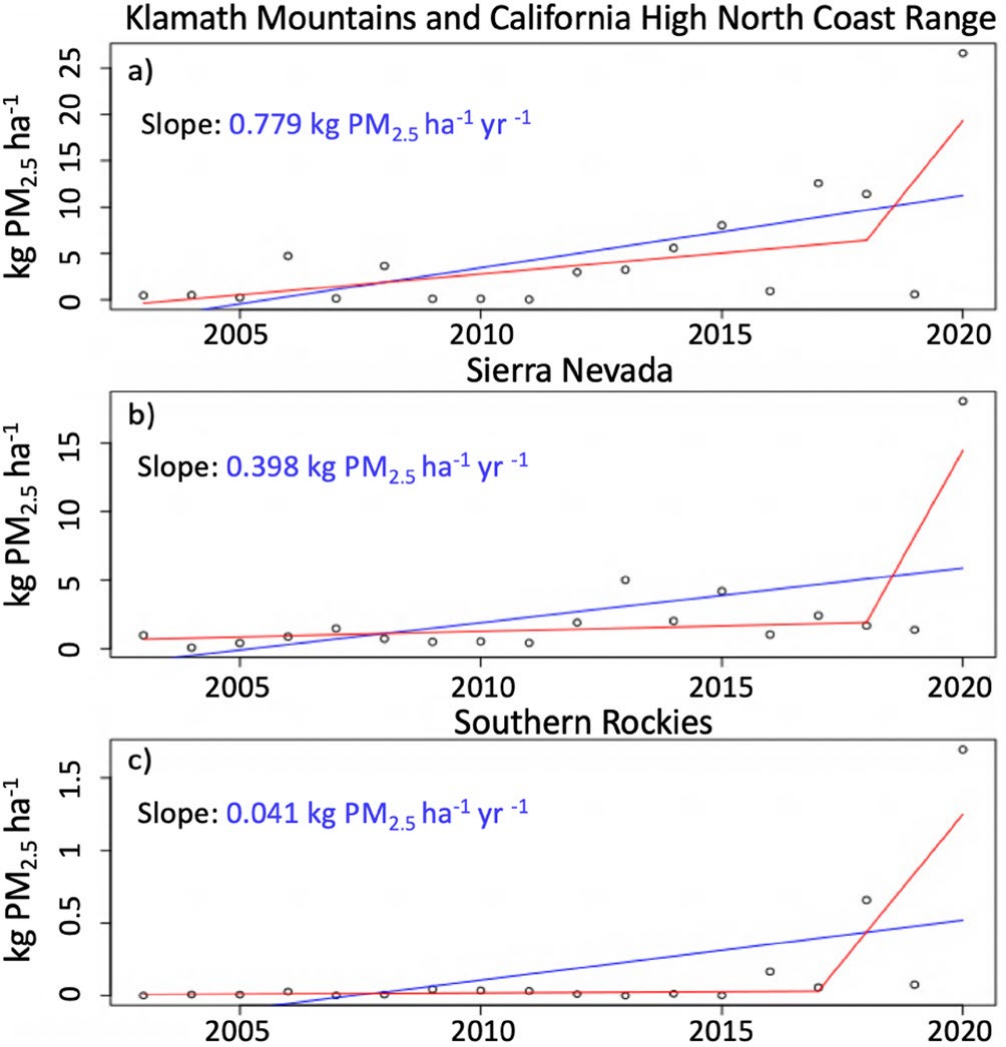
Results of linear (blue) and hinge fit (red) trend analyses for the PM_2.5_ mass injected above the PBL within the (**a**) Klamath Mountains and California High North Coast Range, (**b**) Sierra Nevada, and (**c**) Southern Rockies ecoregions. The slope associated with linear trends is listed in blue.

**Figure 4:**
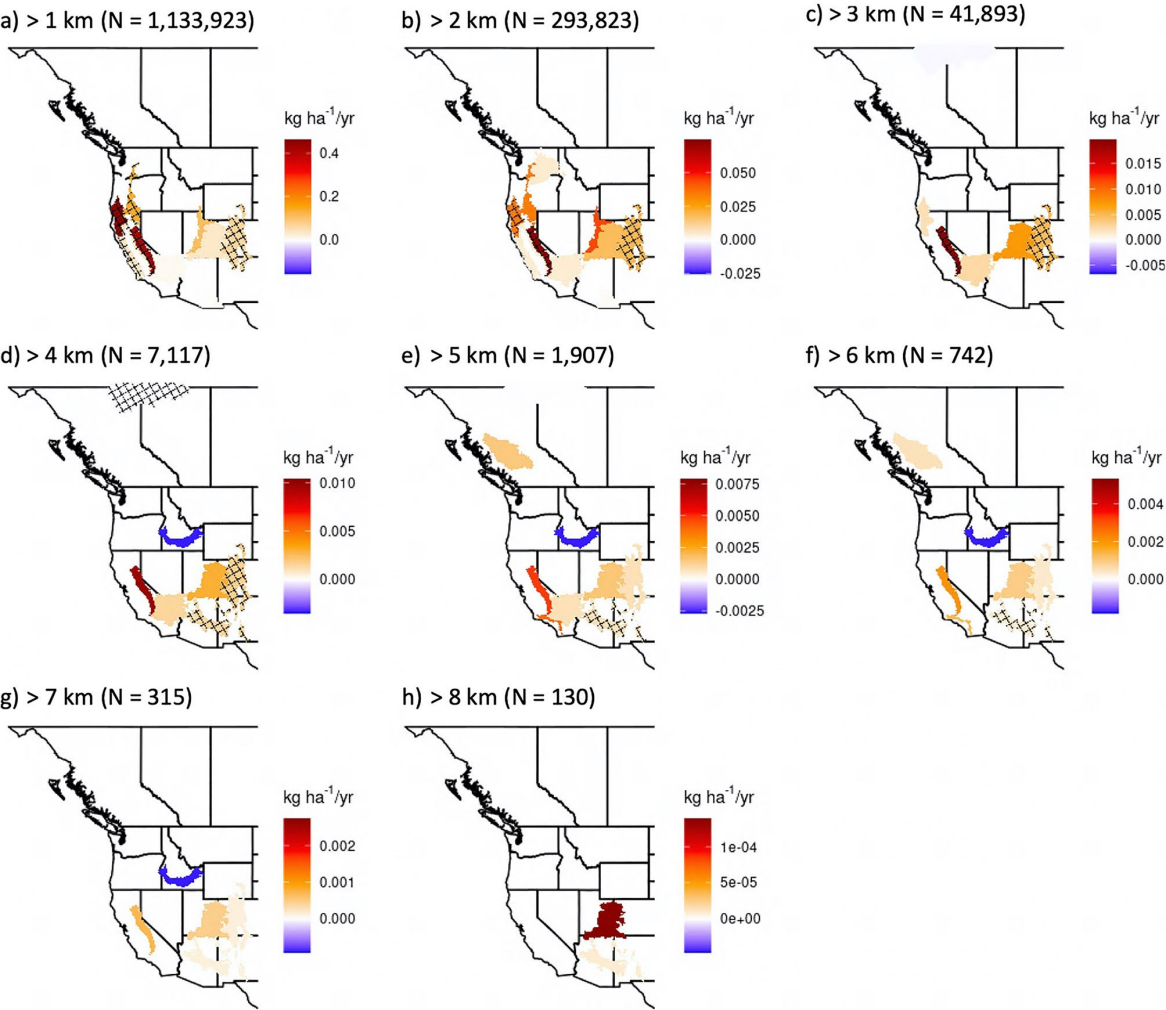
Trends in the PM_2.5_ mass injected above (**a**) 1 km, (**b**) 2 km, (**c**) 3 km, (**d**) 4 km, (**e**) 5 km, (**f**) 6 km, (**g**) 7 km, and (**h**) 8 km, by wildfires during the months of August and September of the years 2003–2020. N-values indicate the number of plumes out of the original ~ 4.6 million that remain above the threshold altitude. Only trends significant at p < 0.1 are displayed, with a crosshatch overlaid on trends with p < 0.05. Note the differences in the ranges represented by the color bars.

An examination of aerosol mass injection indicates trends toward enhanced injection aloft throughout much of the Western US. Trends in aerosol mass injection above or within the PBL (see “PBL estimates from STILT” section in “Methods” section) largely follow total emissions trends (Fig. 2), highlighting wildfire activity in the Klamath Mountains and the Sierra Nevada. This result is noteworthy given the evidence for climate driven enhancements, via atmospheric aridity, to wildfire activity in these regions^18–21^. When limiting timeseries to 2003–2019 (a rather quiet fire year in the Western US), trend analyses further support the presence of positive and significant trends across numerous ecoregions (p < 0.1; p < 0.05—above the PBL in the Sierra Nevada and for all trends in the Klamath Mountains), bolstering confidence in this result given the effect of extreme 2020 values (Fig. 3) on trends fitted to 2003–2020. Altitude-based thresholds highlight significant injection trends up to 7 km altitude for the Sierra Nevada, Southern Rockies, Colorado Plateau, and Arizona—New Mexico mountains (Fig. 4i). A diurnal breakdown of the aforementioned PBL injection trends, based on a 3-h rolling window, indicates that trends are most robust near 18:00 local time (Fig. S1), a result that corresponds to the late afternoon maximum in wildfire activity^54,55^ and the expectation of midday PBL growth. The relevance of enhanced injection above the PBL and at altitudes from 1 to 8 km (Fig. 4) to climate impacts is drawn from recognition of the importance of altitude to extending the aerosol atmospheric lifetime^29,31,32^, enhancing the direct radiative effects of an absorbing aerosol layer^29,68^, and expanding the potential for impact on cloud processes. Recent aircraft-based observations indicate the ability of smoke aerosols to suppress downwind precipitation by acting as a source of cloud condensation nuclei, lowering the average cloud droplet size and staving off initiation of collision-coalescence^12^. Further, enhanced aerosol injection aloft increases the potential for regional-continental scale smoke transport. Thus, Western US and Canadian wildfires will have increasing potential to deteriorate air quality and visibility across much of North America^69^. Concurrent trends toward enhanced aerosol injection within the PBL (Fig. 2b) contribute to elevated risks of wildfire deteriorated air quality at the local scale, a feature of these results that is particularly concerning given a growing Western US population^70^. Hinge fit analyses of aerosol mass injections amplify concern for the potential climate and air quality impacts of Western US wildfires, as results depict the possibility of an emergent acceleration of trends relative to linear trends spanning 2003–2020 (Fig. 3, Fig. S2). While currently statistically insignificant, hinge fit results point toward the possibility of future Western US wildfire impacts to air quality and climate that are in excess of those suggested by a linear fit to 2003–2020. This possible acceleration of trends is in accordance with the extraordinarily large 2020 fire season^71^, and we strongly suspect analysis of the 2021 fire season would lend additional support.

Investigation into the evolution of above PBL emissions as a fraction of total wildfire injected aerosol reveals significant trends toward a growing percentage of emitted mass being injected above the PBL across the Sierra Nevada, Southern Rockies, and Northern Rockies ecoregions. We hypothesize that climate driven trends toward enhanced wildfire activity are responsible for increasing high altitude smoke injections, particularly across the Sierra Nevada and Southern Rockies. Warming enabled trends toward increased high altitude burning throughout the Sierra Nevada and portions of the Rockies^18^ suggest a possible explanation for trends toward preferential smoke injection above the PBL.

### Evaluation against MISR observed plume top heights

**Figure 5:**
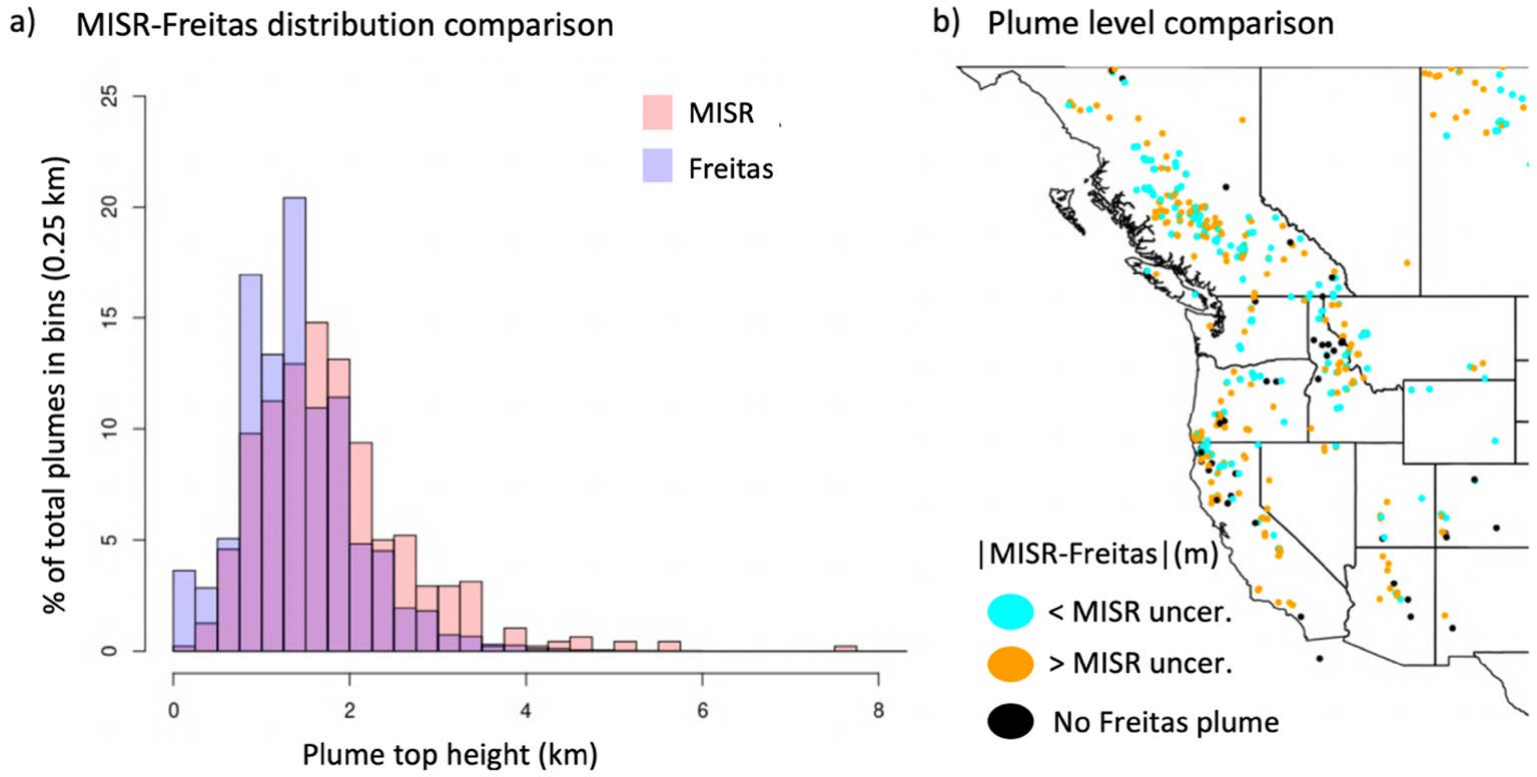
(**a**) A comparison of the distribution of MISR-observed and Freitas-modeled wildfire plume top heights occurring over the Western US and Canada during the months of August and September. (**b**) A map depicting the locations of the MISR-observed plumes used for validation, with color coding used to indicate whether a collocated plume from the modeled climatology agrees within the range of MISR uncertainty. These plumes represent the years 2008–2011 and 2017–2018, reflecting the limited temporal coverage of the MISR plume tops dataset.

Despite the uncertainties associated with generating a regional-decadal scale plume rise climatology (~ 4.6 million plumes), and those inherent to the current state of plume rise modeling^44^, we find that the modeled plumes evaluate well against MISR observed plume top heights when considering the context of previous evaluation efforts^45,56,72^. In an aggregate sense, we see that the distribution of model-derived plume top heights occurring during the late morning MISR overpass window captures much of the distribution of MISR observed plumes (Fig. 5a). While there is a systematic under-estimation of the proportion of high-altitude plumes, this result is in line with prior analyses employing the F2010 model^45^ and is a current reality of the challenges facing physics-based plume top estimates. We find that this model-derived climatology captures the range of MISR observed plume tops, though the numerous high-altitude plumes are proportionally less frequent than what is seen in the observations. As a caveat to this underestimation, it should be noted that the MISR datasets preferentially samples and digitizes larger plumes^51^.

On a plume-by-plume basis, we find a standard deviation of modeled plume top heights from MISR observations of 46.9 m for 425 sets of reasonably collocated plumes (within 30 min and 4 km). Of these 425 sets of collocated plumes, we see that 47.8% of modeled plume tops fall within the ± 500 m observational uncertainty of MISR and that there is little geographic clustering of model uncertainty (Fig. 5b), therefore suggesting consistency of model results across ecoregions. Prior analyses considering smaller spatial and/or temporal domains have reported up to 64% of modeled plume top heights falling within the MISR observational uncertainty^56,72^.

With relevance to above/within PBL injections, 41.4% (176 plumes) of the 425 sets of collocated plumes are found to straddle the PBL, with the modeled plume top above the PBL and the observed plume top within the PBL, or vice versa. In 71% of such cases, the modeled plume top exceeded the PBL, with PBL heights averaging 77.9% of the modeled plume top heights. In terms of the climatological average of the modeled vertical distribution of smoke below plume top, ~ 78% of the PM_2.5_ associated with these modeled plumes is being injected within the PBL. Given that the MISR observed plume top for these cases was contained within the PBL, this equates to an average model underestimate of the PM_2.5_ injected within the PBL of ~ 22% for such cases. Similarly, if it is assumed that the smoke is distributed evenly throughout a MISR observed plume and that the retrieved plume top heights should follow a normal distribution for any individual plume, an average of ~ 77% of the injected PM_2.5_ is contained within the PBL for the 29% of cases in which the MISR observation exceeded the PBL and the modeled plume top did not. Based on the findings of Val Martin et al.^51^, this assumption of a normal distribution for MISR retrieved heights is reasonable in many cases, though a lognormal distribution is likely more common. As such, it is reasonable to expect that > 77% of the PM_2.5_ associated with these MISR observed plumes is retained within the PBL, enhancing the agreement with modeled injections. Overall, this analysis supports the idea of largely cancelling over/underestimation of PM_2.5_ injection relative to the PBL, though given the greater frequency of modeled plume tops exceeding the PBL when MISR observations do not, it appears likely that the net effect is a modest overestimation of above PBL PM_2.5_ injection.

## Discussion

We find that trends toward enhanced wildfire activity correspond to elevated wildfire plume top heights and aerosol injection aloft for the majority of mountainous ecoregions across the Western US. August–September plume top maxima (Fig. 1a) and PM_2.5_ flux-weighted average plume tops (Fig. 1b) exhibit statistically significant increasing trends across many Western U.S. ecoregions. These trends were collocated with increases in wildfire emissions (Fig. 2c), most notably across the Sierra Nevada ecoregion. Recast in terms of the aerosol mass injected, we find that the spatial variability of injection trends above and within the PBL (Fig. 2a,b) is largely explained by variability in wildfire emissions trends, albeit the Sierra Nevada and portions of the Rockies demonstrate a shift towards preferential smoke injections above the PBL. We hypothesize that the Sierra Nevada and Southern Rockies are exhibiting trends toward a greater fraction of smoke being emitted above the PBL due to climate enabled enhancements in high altitude burning^18,73^. Furthermore, there is a potentially emergent acceleration of flux-weighted average plume top, aerosol mass injection, and pyroCb trends over recent years (2017/2018–2020). Enhanced high-altitude injections of smoke aerosol mass (Fig. 4) suggests evolution of the vertical distribution of wildfire emitted aerosols in response to increasingly intense wildfire activity. In light of the climate change—wildfire connection across the Western US^14–24^, we suspect a causal chain that, in part, implicates climate change in elevated wildfire plume top heights and enhanced aerosol injection aloft.

Given the link between aerosol injection altitude and the corresponding air quality impacts, our findings suggest that wildfire activity in the Western US presents a growing risk in terms of long-range smoke transport and air quality degradation. Concurrent upward trends in the mass of biomass burning aerosols injected above and within the PBL present a threat to human health across local to continental scales. Recent wildfire activity stands testament to this, with the 2020 fire season likely producing thousands of smoke-related deaths^71^, and satellite imagery suggesting continental scale smoke transport akin to the documented presence of Western US/Canadian wildfire smoke in New York City in August of 2018^74^.

From a climate perspective, these results point to Western US wildfire activity as becoming increasingly relevant to regional climate. Injection of a greater aerosol mass at higher altitudes, potentially into the upper troposphere—lower stratosphere, corresponds to an enhanced direct radiative effect given longer atmospheric lifetimes and increased absorption efficacy^9,29,31,32,68^. While less understood, there are also potential indirect effects related to aerosol-cloud microphysical interactions^12,13,34^. Though speculative, the potential for wildfire-derived aerosols to act as cloud condensation nuclei^13^ and stave off initiation of rainfall^12,13^ is further cause for concern given the risk of a positive feedback on wildfire activity.

As caveats to the results presented within this study, we note that due to the multi-decadal timeframe considered and the associated computational cost, the model framework neglects the possibility of multi-core plume updrafts within an active fire^75^, as well as fire-atmosphere and aerosol-PBL feedbacks that can alter the PBL height and aerosol mass fraction contained within the PBL^76–78^. Additionally, we do acknowledge that vegetation type produces variability in the heat release per unit dry matter burned by wildfires. However, this heat release remains relatively constant between 16 and 22 MJ/kg as shown in pervious literature^79^. Furthermore, the F2010 model exhibits relatively low sensitivity to heat flux inputs^53^. Therefore, we suspect that the assumed heat release constant of 18.6 MJ/kg has not skewed the results presented here.

While the questions addressed herein provide a critical initial view of how the vertical distribution of wildfire emitted aerosols has evolved over the Western US and Canada over the past two decades, the presented hinge fit results suggest that this topic will need to be revisited in future studies. Further development of wildfire plume rise modeling and the inclusion of additional years of data should provide for an improved understanding of trends in wildfire aerosol injection. Furthermore, given global variability in wildfire activity^80^, it is apparent that investigation into plume height trends at the global scale would better elucidate the potential climate impacts of increasingly intense wildfire activity^14–18^. Such an investigation would be particularly valuable should plume height trends be considered within a radiative modelling framework. An expanded understanding of the evolving vertical distribution of wildfire emitted aerosols would hold direct relevance to air-quality and climate change projections, expression of wildfire emissions in near-field modeling efforts, and the accuracy of aerosol layer height assumptions required for satellite retrievals of absorbing aerosols.

## Methods

### Data

The spatial information, temporal information, and usage of datasets included in this study are presented in Table 1. Individual dataset descriptions can be found in Supplemental Information (SI) Sect. S2.

**Table 1:** A description of the data products used.

Product name	Spatial info	Temporal info	Usage
QFED PM_2.5_ emissions	0.1°, CONUS/CA	Daily, 2003–2020	Heat flux derivation, PM_2.5_ injections
MCD64A1 version 6	500 m, CONUS/CA	Daily, 2003–2020	Active burn area
GFED 4.1 s	0.25°, CONUS/CA	2003–2020	Temporal downscaling
MISR plume heights (MINX v.4)	1.1 km horizontal resolution, 500 m vertical uncertainty	Late morning overpass, 2008–2011, 2017–2018	Model evaluation
TDL U.S. and Canada Surface Hourly Observations^81^	1258 sites, CONUS/CA	Hourly, 2003–2020	Surface WRF evaluation
Rawinsonde data^82^	24 sites, CONUS/CA	00/12 UTC, 2003–2020	Upper air WRF evaluation
Climate Forecast System (Reanalysis & v2)^83^	0.5°, 40 vertical levels	6-h, 2003–2020	WRF initial and boundary conditions
USGS land use types	~ 1 km	Static	WRF input

The spatial and temporal information reflects the regions and years used in this study. Geographic acronyms: Continental United States (CONUS), Canada (CA).

*All datasets subset to West of − 100° E longitude.

### Models

#### Freitas et al. plume rise model

The F2010 model is a 1-dimensional, physics-based model that relies on the vertical momentum equation, the first law of thermodynamics, and mass continuity for water phases. Processes related to lateral and dynamic entrainment are also parameterized within this model. The F2010 model estimates the wildfire plume top height as a function of wildfire heat flux density, active burn area, and environmental conditions ingested from an atmospheric transport model. By iteratively solving for a steady state plume rise solution, the F2010 model is able to approximate the plume top height and a thermodynamic profile of the final plume at 100 m vertical resolution. The plume top height is defined as the altitude at which the plume is neutrally buoyant, and is approximated as a vertical velocity < 1 m/s. Entrainment coefficients, output as part of the thermodynamic profile, can be used to estimate the vertical profile of aerosol detrainment below plume top when it is assumed that mass is conserved within the column for a steady state solution.

While uncertainties remain in the model, F2010 was selected for its adherence to a physical basis and its ability to account for latent heat release by cloud microphysical processes^47,53^. Empirical plume rise schemes neglecting latent heat release through condensation and freezing are thus at a disadvantage relative to F2010. Evaluating the performance of the F2010 model is difficult given limited observations and the relatively poor constraint on wildfire heat flux and active burn area inputs. A breakdown of errors due to input uncertainty relative to the model structure is unknown at this time. In an evaluation of the F2010 model against MISR plume top observations, Val Martin et al.^45^ take a range of approaches to model input, ultimately finding a maximum correlation strength to the observations of ~ 0.3 and a consistent underestimation of the dynamic range of plume top heights. Ke et al.^56^ noted that 64% of their simulations using the F2010 model generated plume top heights within the MISR uncertainty of ± 500 m, a result comparable to the 63% value associated with a similar evaluation of the Sofiev et al.^72^ plume rise formulation. In this study, given the number of plumes to be modelled (~ 4.6 million), explicit simulation of individual wildfire plumes using a coupled atmosphere—fire spread model (e.g. WRF-Sfire)^84^ was not computationally feasible.

For this study, we supplied the F2010 model with environmental conditions from nested Weather Research and Forecasting (WRF)^85^ model output, and wildfire inputs generated through a combination of wildfire PM_2.5_ emissions estimates^61^, burned area estimates (MCD64A1)^86^, and sub-daily temporal downscaling profiles based on satellite active fire detections^54,80^.

##### WRF—atmospheric inputs

The environmental condition inputs to the F2010 model were supplied by a regional-decadal scale WRF simulation. For this study, we used an updated version of the Freitas model described and evaluated by Mallia et al.^57^, which can ingest WRF input files directly. The WRF simulations generated for this study used a nested domain setup where a 4 km resolution domain covered most of the Western US and was nested within a 12 km resolution domain covering western North America (Fig. S3). WRF simulations were reinitialized weekly using initial and boundary conditions from the Climate Forecast System Reanalysis^83^. The physical parameterizations used in these WRF simulations can be found in SI Sect. S3 and were based on configurations recommended by Mallia et al.^87^. Simulated plume top heights were not sensitive to the resolution of WRF domains as seen in the sensitivity analysis carried out in SI Sect. S3.

To evaluate the performance of these WRF simulations for surface winds, temperature, and humidity, we used METSTAT, a software package designed to evaluate meteorological inputs to air quality models^88^. Using METSTAT, WRF gridded variables were interpolated to the locations of surface meteorological observations from 1258 sites in the TDL U.S. and Canada Surface Hourly Observations dataset^81^ to assess the performance of our WRF model simulations. We calculate the bias and root mean square error (RMSE) for surface wind speed, wind direction, temperature, and humidity (Table 2). When compared to benchmark values^89^, the WRF meteorology generated here outperforms the suggested benchmarks for surface wind speed bias, wind speed RMSE, wind direction bias, temperature bias, and humidity bias. Emery et al.^89^ do not present RMSE benchmarks for surface wind direction, temperature, or humidity. The bias and RMSE of surface WRF variables compare favorably to those presented in related work by Mallia et al.^87^, though given overlap with the WRF setup used by Mallia et al.^87^, this likely owes to greater averaging of model errors over space and time.

**Table 2:** Bias and root mean square error (RMSE) values associated with evaluation of the generated WRF meteorology (domain1) against surface observations and observations aloft.

Variable	Bias	RMSE
Surface temperature (K)	0.098	0.732
Potential temperature aloft (K)	−0.011	2.33
Surface wind speed (m/s)	−0.314	0.372
Wind speed aloft (m/s)	−1.510	4.442
Surface wind direction (deg.)	2.666	4.453
Wind direction aloft (deg.)	−5.098	65.206
Specific humidity (g/kg)	−0.235	0.511

The upper-air evaluation of WRF wind speed, wind direction, and potential temperature was carried out using data from rawinsonde launches at 24 sites throughout the Western US and Canada^82^. For each balloon launch, the vertical profiles of U, V, and potential temperature were extracted from the corresponding 12 km WRF grid cell at the time of the balloon launch. Horizontal winds simulated by WRF were then converted to wind speeds and wind directions, while vertical profiles of rawinsonde data were interpolated by pressure to match WRF vertical levels. To focus the evaluation on simulated upper air values and to avoid the effects of greater turbulence within the PBL, evaluation was limited to vertical levels situated above the simulated PBL height. Furthermore, our evaluation was only performed for rawinsonde observations at which the balloon had been advected less than 4 km from the original launch site. This 4 km limit was selected to balance between the need for ample observations to evaluate WRF output and the fact that the quality of the evaluation would be degraded when using observations of a balloon advected into adjacent WRF grid cells. The bias and RMSE of wind speed, wind direction, and potential temperature were calculated (Table 2).

##### Wildfire inputs

Gridded QFED PM_2.5_ emissions (0.1° resolution)^61^ are provided at daily resolution for four biome types (extratropical forests, grasslands, savannahs, and tropical forests) and were converted to a heat flux dataset using PM_2.5_ emissions factors (PM_2.5_ emitted/kg dry matter burned)^90^ and assuming that the heat release per unit dry matter burned is 18.6 MJ/kg^57,91^. This conversion is depicted in Eq. (1), where ${F}_{heat}$ is the gridded heat flux, and ${PM}_{2.5,i}$ and ${EF}_{i}$ are the gridded PM_2.5_ flux and emission factor associated with biome type $i$, respectively.

1 $${F}_{heat}=18.6 \times \sum_{i=1}^{n=4}{(PM}_{2.5,i}\times {EF}_{i}).$$

Heat flux datasets were then overlaid with daily burned area polygons from Moderate Resolution Imaging Spectroradiometer (MODIS) collection 6 to identify probable linkages between wildfire heat flux and satellite observed burned area. Since QFED emissions are based on MODIS observed thermal hotspots (MOD14A1/MYD14A1 product), the literature suggests an expectation of collocated burned area and wildfire heat fluxes within a ± 8 day window (algorithm nominal uncertainty^92^), with the majority of collocations occurring within ± 1 day^86,93^. Given this temporal window, we linked wildfire heat fluxes to burned area polygons using three complementary approaches that account for the temporal uncertainty in wildfire burned area observations and the idea of a directional fire spread. Specifics regarding this process are provided in SI Sect. S4, with the relevant methods depicted within a detailed visual (Fig. S5).

After developing relationships between gridded heat fluxes and burned area, we assumed that each cluster of continuous burned area polygons would produce its own plume. Within a given QFED grid cell, daily heat fluxes were then attributed to each intersecting cluster of burned area polygons based on the fraction of the total burned area within the QFED grid cell comprised by the burned area cluster. Burned area clusters intersecting multiple grid cells with non-zero heat fluxes were linked to heat fluxes from each grid cell based on the area of overlap within that grid cell. Ultimately, the heat flux value attributed to each burned area cluster was divided by the area of that cluster to estimate the heat flux density.

In total, 78% of the MODIS burned area was paired with a gridded heat flux, with this value increasing to 89% when considering only burned area polygons that intersect a grid cell with non-zero heat flux within the ± 8 day window. Similarly, 91% of QFED derived heat fluxes were linked to burned area polygons. Possible explanations for the imperfect collocation of heat fluxes and MODIS burned area polygons could stem from clouds obscuring satellite observation and differences between the spatial resolution of the MCD64A1 burned area product and the MOD14A1 thermal hotspot product used by QFED. Generally, years characterized by greater wildfire activity present greater collocation of heat fluxes and burned area (Table S2). We hypothesize that this relationship is the result of resolution limitations to observation, such that more intense wildfire activity is more readily observed by both the MCD64A1 and MOD14A1 products, increasing the odds of collocated observations.

Temporal downscaling from daily to 3-h emissions and burned area was performed using diurnal cycles of wildfire activity based on gridded (0.25° resolution) and monthly aggregated satellite active fire detections provided by the Global Fire Emissions Database (GFED)^54,80^. For each set of plume inputs, the centroid of the associated burned area cluster was referenced to the GFED grid, and the relevant diurnal cycle was applied to both the heat flux and active burn area (such that the heat flux density remained unchanged). The resultant values were assumed to apply to each hour within the 3-h window, meaning additional plume top/thermodynamic variability within any 3-h downscaling window would strictly be a result of varied meteorological conditions.

#### Entrainment–mass conservation

To estimate the vertical distribution of smoke detrainment below plume top, thermodynamic F2010 outputs for each plume were subject to vertical level-by-level mass conservation calculations following Eq. (2), where ${D}_{mass, Z}$ is the mass of in plume air detrained from vertical level $Z$, ${E}_{mass,Z}$ is the mass of environmental air entrained into the plume at vertical level $Z$, and the remaining terms characterize the vertical flux of mass into the layer from the layer below and the vertical flux of mass out of the layer to the layer above, respectively (left-to-right). The vertical mass flux between any two layers was taken to be a product of their mean density ($\overline{\rho }$), mean vertical velocity ($\overline{W }$), and mean plume horizontal area ($\overline{A }$), ideally characterizing the interface between layers. At the plume top, it was assumed that the vertical flux to the above layer is zero, such that any remaining vertical mass transport is redirected as detrainment within the uppermost layer.

2 $${D}_{mass, Z}={E}_{mass,Z}+\left(\overline{{\rho }_{Z, Z-1}}\right)\left(\overline{{W }_{Z, Z-1}}\right)\left(\overline{{A }_{Z, Z-1}}\right)-\left(\overline{{\rho }_{Z, Z+1}}\right)\left(\overline{{W }_{Z, Z+1}}\right)(\overline{{A }_{Z, Z+1}})$$

To express level-by-level detrainment values in terms of a vertical distribution of wildfire emitted aerosol, vertical profiles of detrainment were linearly interpolated from 100 m vertical resolution to 10 m vertical resolution, and then normalized. For interpolation, it is assumed that the detrainment at the surface and plume top boundaries is equal to 0. Resultant profiles effectively describe the fraction of total plume aerosol emissions that are injected at each 10 m vertical level.

#### PBL estimates from STILT

STILT calculates the PBL height using a modified Richardson number method that is applicable under stable, neutral, and unstable conditions^62,94^. Here, STILT is used to estimate the PBL height relevant to each modeled plume rise by initializing a model receptor on the centroid of the active burn area at the time of the plume rise. PBL heights are then extracted from the STILT output and linked with the plume climatology, allowing identification of penetrative plumes and quantification of the aerosol mass injected above the PBL. Meteorological inputs to STILT are sourced from the nested WRF simulations described above.

### Trend analyses

Trend analyses aimed at understanding the evolution of plume top heights and the vertical distribution of wildfire generated aerosol mass, were performed for US level 3 ecoregions^66^ and Canadian ecoprovinces^67^. For each ecoregion/ecoprovince West of − 100° E, the annual maximum plume top height, the PM_2.5_ flux-weighted average plume top height, the PM_2.5_ mass injected relative to threshold altitudes (> 18 km, > PBL, < PBL), and the total emissions were subjected to linear regression. Statistical significance was addressed via 100,000 bootstrapped repetitions. Each linear regression was reapplied to 100,000 resampled versions of the annually aggregated values, allowing replacement of selected samples, to determine if the regression to the actual data was distinguishable from the scatter produced by resampling.

Hinge fitting, or change point analysis, was also used to elucidate potential emergent trend accelerations over recent years. Using the statistical analysis software R^95^, the “chngpt” package^96^ was used to fit 2-segmented linear trends to PM_2.5_ flux-weighted average plume top height, PM_2.5_ mass injection relative to the PBL, and PM_2.5_ emissions timeseries. Significant hinge-points were selected using a bootstrapping procedure that identifies the optimal hinge, with an associated p-value based on the quality of model fit. Hinge fitting has previously been used to compute climate normals amidst a changing climate^97^.

### Comparison to MISR plume top heights

MISR derived plume top heights were used to evaluate the performance of the F2010 model by comparing the distribution of modeled and observed plume top heights. Additionally, a plume-by-plume analysis was performed, which considers the deviation between modelled and observed plume tops in the context of instrument uncertainty. From the archived plumes maintained as part of the MISR Plume Heights Project 2, we found 480 plumes with a quality flag of “good” or “fair” within western North America (Fig. 5b). These observed plumes characterize the years 2008–2011 and 2017–2018. Using bin widths of 250 m, we developed a histogram comparing these 480 observed plumes to the model-derived late morning plumes (corresponding to MISR overpass times) spanning the same years (251,262 plumes; Fig. 3a). Distribution level comparisons between F2010 model output and MISR observations have previously identified a strong sensitivity to model inputs and a tendency for the model to underestimate the range of observed plume top heights^45^.

On a plume-by-plume scale, we found 425 MISR observed plumes within 30-min and 4 km of a model-derived plume. A cutoff distance of 4 km was used to compromise between the importance of local meteorology to plume top heights and the uncertainty in plume location as a result of using burned area centroids to denote plume locations. In cases when hourly resolution model outputs result in a plume top estimate both before and after the MISR overpass time, modeled plume top heights are temporally interpolated to the timing of the MISR overpass. Analysis of matched sets of plumes focusses on the standard deviation of modeled plume top heights from their observed counterparts and the fraction of modeled plumes falling within the MISR observational uncertainty.

## Supplementary Information

Supplementary Information.

## Data Availability

The model-derived wildfire plume data generated and analyzed during the current study are available from the corresponding author on reasonable request. MISR observed wildfire plume top data are available at the following web address: https://misr.jpl.nasa.gov/getData/accessData/MisrMinxPlumes2/. QFED wildfire emissions estimates, GFED wildfire temporal downscaling datasets, and MCD64A1 wildfire burned area polygons are available from at https://portal.nccs.nasa.gov/datashare/iesa/aerosol/emissions/QFED/v2.5r1/0.1/QFED/, https://globalfiredata.org/pages/data/, and University of Maryland via ftp, respectively.
