# Supplementary material for: Wildfire plumes in the Western US are reaching greater heights and injecting more aerosols aloft as wildfire activity intensifies
Source: Sci Rep. 2022 Jul 20;12:12400. doi: 10.1038/s41598-022-16607-3 (PMC9300699; doi:10.1038/s41598-022-16607-3)
Supplement: Supplementary file 1 — Supplementary Information. [file 41598_2022_16607_MOESM1_ESM.docx]

**Supplemental Information**

**S1. Supplemental injection trends**

| **Table S1.** A table indicating the number of simulated plumes per year (2003-2020) during the months of August and September for select ecoregions. | **Eastern Cascades** | **Cascades** | **Mojave Basin and Range** | **Arizona – New Mexico Mts.** | **Colorado Plateau** | **Wasatch-Uinta Mts.** | **Southern Rockies** | **Klamath Mts./ Cali. High N. Coast Range** | **Central California Foothills & Mts.** | **Sierra Nevada** | **Ecoregion** |
| --- | --- | --- | --- | --- | --- | --- | --- | --- | --- | --- | --- |
|  | 1,786 | 5,230 | 0 | 486 | 530 | 788 | 254 | 1,892 | 2,073 | 5,245 | **2003** |
|  | 258 | 499 | 320 | 791 | 1,558 | 846 | 594 | 2,006 | 1,879 | 492 | **2004** |
|  | 57 | 144 | 1,050 | 384 | 414 | 312 | 831 | 3,222 | 828 | 2,593 | **2005** |
|  | 180 | 1,617 | 120 | 285 | 0 | 300 | 1,095 | 23,166 | 258 | 4,410 | **2006** |
|  | 1,152 | 1,314 | 0 | 1,005 | 840 | 414 | 237 | 735 | 2,772 | 4,224 | **2007** |
|  | 1,311 | 6,645 | 0 | 255 | 852 | 270 | 336 | 17,727 | 1,287 | 4,062 | **2008** |
|  | 1,140 | 7,922 | 0 | 7,461 | 1,272 | 1,887 | 1,077 | 924 | 2,922 | 1,734 | **2009** |
|  | 1,385 | 1,364 | 0 | 1,053 | 819 | 5,253 | 584 | 1,254 | 734 | 2,568 | **2010** |
|  | 1,965 | 3,810 | 597 | 2,091 | 312 | 135 | 660 | 96 | 1,347 | 1,854 | **2011** |
|  | 14,655 | 10,287 | 924 | 903 | 279 | 216 | 870 | 14,784 | 2,364 | 7,920 | **2012** |
|  | 1,830 | 696 | 0 | 1,026 | 0 | 384 | 0 | 12,303 | 1,419 | 25,470 | **2013** |
|  | 2,364 | 5,967 | 0 | 1,176 | 39 | 0 | 192 | 24,930 | 216 | 9,539 | **2014** |
|  | 5,397 | 5,607 | 648 | 717 | 0 | 528 | 147 | 32,595 | 5,688 | 18,552 | **2015** |
|  | 747 | 0 | 480 | 291 | 666 | 2,718 | 3,093 | 3,525 | 16,860 | 2,958 | **2016** |
|  | 3,571 | 28,844 | 122 | 130 | 346 | 975 | 1,118 | 40,469 | 632 | 7,806 | **2017** |
|  | 8,053 | 4,915 | 0 | 2,039 | 2,602 | 8,900 | 8,208 | 54,299 | 8,435 | 8,238 | **2018** |
|  | 732 | 747 | 0 | 7,053 | 732 | 654 | 792 | 1,137 | 408 | 5,046 | **2019** |
|  | 11,445 | 42,670 | 1,716 | 9,153 | 8,907 | 6,075 | 22,968 | 106,518 | 59,685 | 75,447 | **2020** |
| **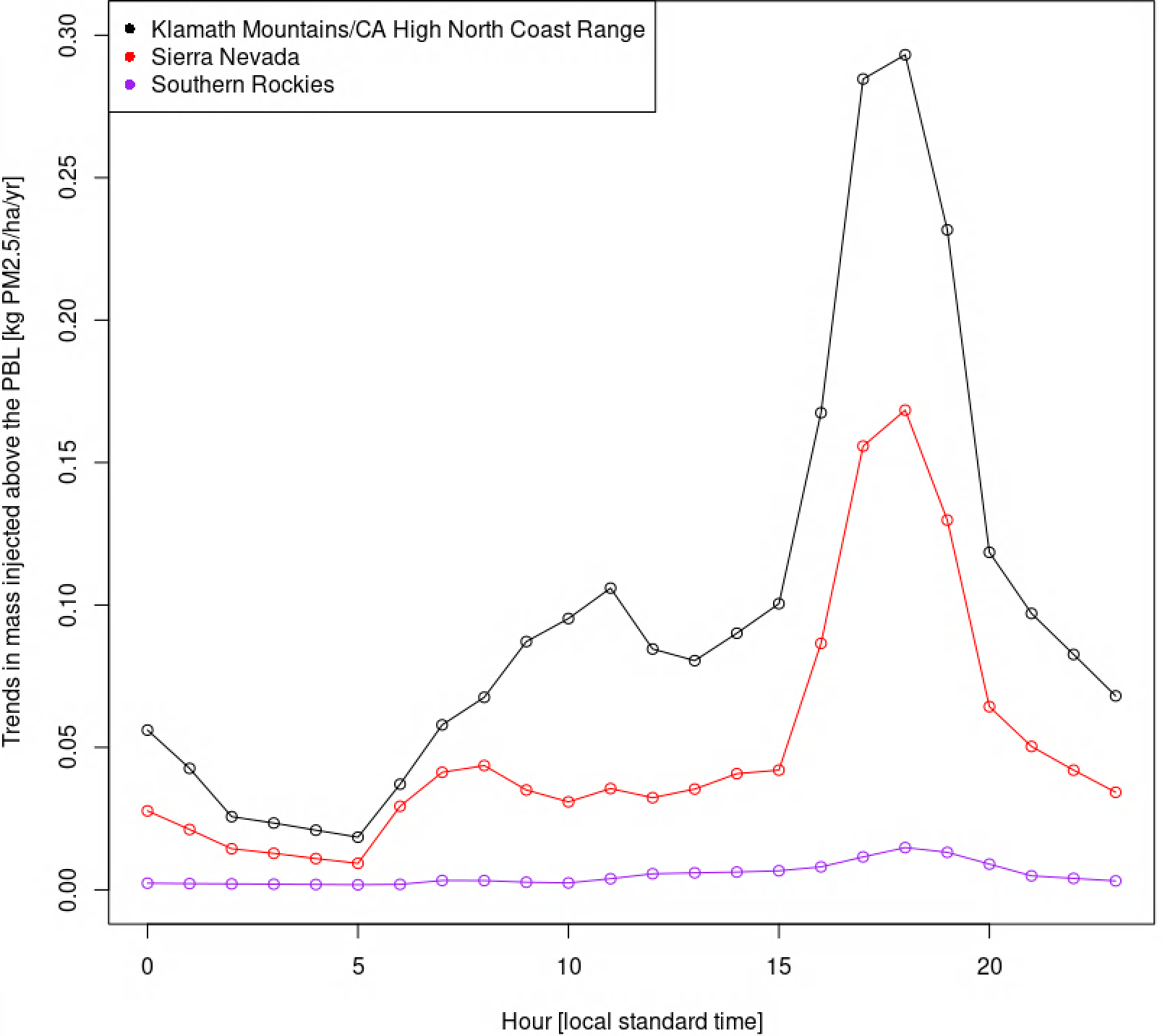** | | | | | | | | | | | |
| **Figure S1.** Results for the diurnal cycle of above PBL mass injection trends for the Klamath Mountains and California High North Coast Range (black), Sierra Nevada (red), and Southern Rockies (purple) ecoregions. Based on a 3-hour rolling window. | | | | | | | | | | | |

| **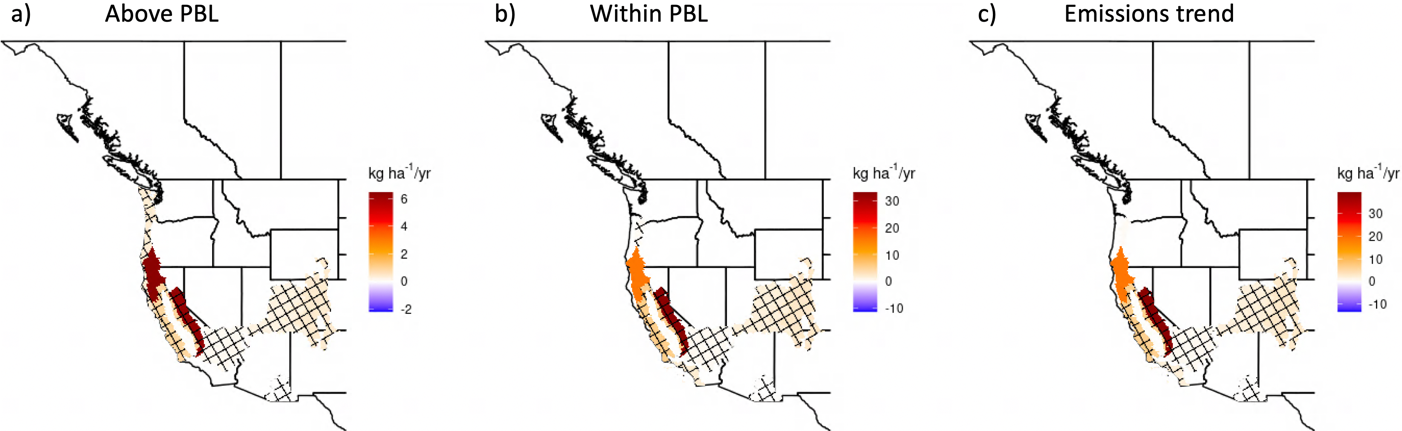** |
| --- |
| **Figure S2.** Results for hinge fit trend analyses (to the right of the hinge) of the PM_2.5_ mass **(a)** injected above the PBL, **(b)** injected within the PBL, and **(c)** emitted by wildfires for Western US ecoregions and Canadian ecoprovinces during August and September. Only results for ecoregions/ecoprovinces with a linear fit trend significant at p < 0.1 and a hinge point significant at p < 0.1 are depicted. Ecoregions/ecoprovinces with a hinge point significant at p < 0.05 are displayed with an overlaid crosshatch. Note the differences in the ranges represented by the color bars in (a)-(c). |

**S2. Dataset descriptions**

QFED PM2.5 emissions

QFED is a near real time, daily, 0.1-degree resolution fire emissions inventory. QFED emissions are based on the top-down fire radiative power approach (Darmenov and da Silva, 2015). We use QFED v2.5r1 PM_2.5_ emissions for 4 biome types for August-September of 2003-2020 to work backward to a gridded heat flux dataset. Heat flux values are then used for development of inputs to the F2010 model.

MCD64A1 v006

MCD64A1 is a burned area dataset based on remote sensing of the earth’s surface by the MODIS instrument onboard the Terra and Aqua satellites. A burn sensitive vegetation index is used for thresholding of land surface reflectance to identify burned tiles at horizontal and temporal resolutions of 500 m and daily, respectively. We use all MCD64A1 burned area polygons within the Western US and Canada (west of -100°E and south of 60°N) for 24 July – 8 October of 2003-2020.

GFED 4.1s

GFED is a global wildfire emissions inventory based on a hybrid bottom-up and top-down approach to emissions estimates, in which the majority of emissions are estimate from bottom-up information of fire area burned and vegetation type, while emissions for small fires are estimated using top-down satellite active fire detections. Specifically, we use GFED provided data for temporal downscaling of emissions from daily to 3-hour intervals based on climatological data of active fire detections at 0.25° spatial resolution. Downscaling profiles are consistent over a given month, but vary from one year to the next.

MISR plume heights (MINX v.4)

The MISR Plume Height Project 2 provides a dataset of digitized MISR observed plume top heights that can be found at the following URL: <https://misr.jpl.nasa.gov/getData/accessData/MisrMinxPlumes2/>. With a maximum uncertainty of 500 m, plume top heights are estimated by means of a stereo-height derivation that leverages multiple view angles and identifies the level of maximum reflectance contrast. In addition to providing a plume top height estimate, the dataset further contains geospatial/temporal information and quality flags to allow for sub-setting of the data. A challenge of the MISR plume tops dataset is that it is limited to observing plumes during the late morning overpass time, and thus does not capture the diurnal variability of wildfire plume top heights.

**S3. WRF physical parameterizations and sensitivity of the F2010 model to WRF resolution**

The physical parameterizations employed for WRF simulations included the Rapid Radiative Transfer Model (RRTMG) for short and longwave radiation (Iacono et al., 2008), the NOAH land-surface model (Chen and Dudhia, 2001), the Mellor – Yamada – Janjic turbulent kinetic energy scheme for PBL physics (Mellor and Yamada, 1974; Mellor and Yamada, 1982; Janjić, 2002), and the Grell–Devenyi ensemble scheme for parameterized cumulus (Grell and Devenyi, 2002; Lin et al., 1983). The WRF setup further included one-way domain nesting, neglected data assimilation options (fdda), and used 44 vertical levels spanning from the surface to 10 hPa.

1. M.J. Iacono, J.S. Delamere, E.J. Mlawer, M.W. Shephard, S.A. Clough, W.D. Collins, Radiative forcing by long-lived greenhouse gases: Calculations with the AER radiative transfer models. *J. Geophys. Res.* **113**, D13103 (2008).
2. F. Chen, J. Dudhia, Coupling an advanced land-surface–hydrology model with the Penn State–NCAR MM5 modeling system. Part I: Model implementation and sensitivity. *Mon. Weather Rev.* **129**, 569–586 (2001).
3. G.L Mellor, T. Yamada, A hierarchy of turbulence closure models for planetary boundary layers. *J. Atmos. Sci.* **31**, 1791-1806 (1974).
4. G.L. Mellor, T. Yamada, Development of a turbulence closure model for geophysical fluid problems. *Rev. Geophys. Space Phys.* **20**, 851-875 (1982).
5. Z. Janjic ́, Nonsingular implementation of the Mellor–Yamada level 2.5 scheme in the NCEP meso model. *NCEP Office Note.* No. 437, 60 (2002).
6. G.A. Grell, D. Devenyi, A generalized approach to parameterizing convection combining ensemble and data assimilation techniques. *Geophys. Res. Lett.* **29**, 1693–1696 (2002).
7. Y.-L. Lin, F.D. Farley, H.D. Orville, Bulk parameterization of the snow field in a cloud model. *J. Appl. Meter.* **22**, 1065-1092 (1983).

| 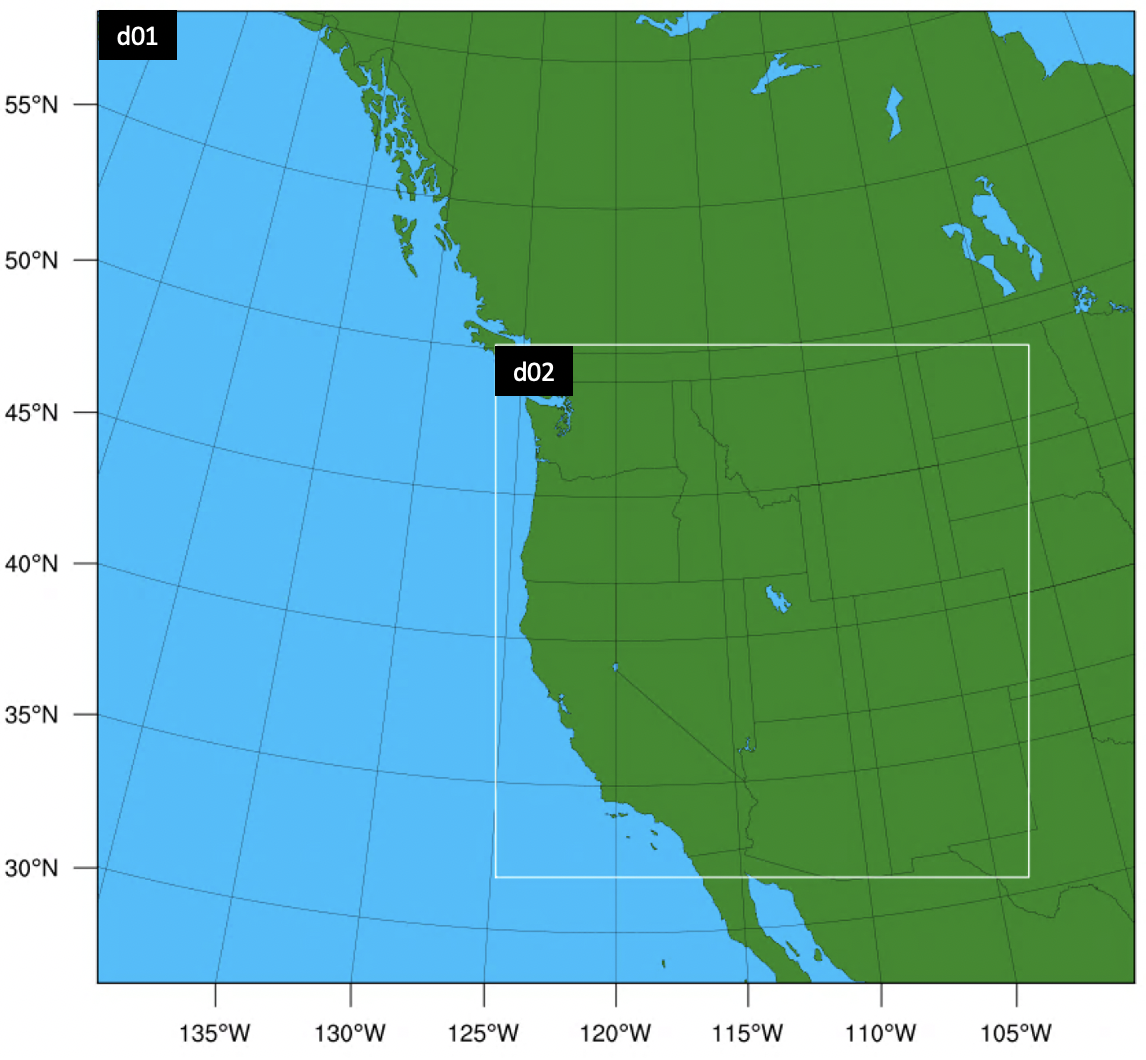 |
| --- |
| **Figure S3.** WRF domain configuration, where d02 has a 4 km spatial resolution and the larger parent domain (d01) has 12 km spatial resolution. This map was produced using the plotgrids_new.ncl script within the WRF Pre-Processing System of WRF version 4.2. This script can be found on GitHub at <https://github.com/wrf-model/WPS/blob/master/util/plotgrids_new.ncl>. |

To understand the variability in simulated plume top heights as a result of the spatial resolution of input meteorology, we re-ran all of the 2018 plumes that reside within d02 (n = 182,755) using the 12 km meteorology of d01. A comparison of simulated plume top heights using d01 (12 km) and d02 (4 km) meteorology demonstrates strong agreement, especially in plume top heights exceeding 500 m (fig. S4a). The distribution of plume top height differences (4km met. – 12 km met., fig. S4b) further confirms that simulated plume top heights are largely insensitive to the resolution of meteorological inputs.

| 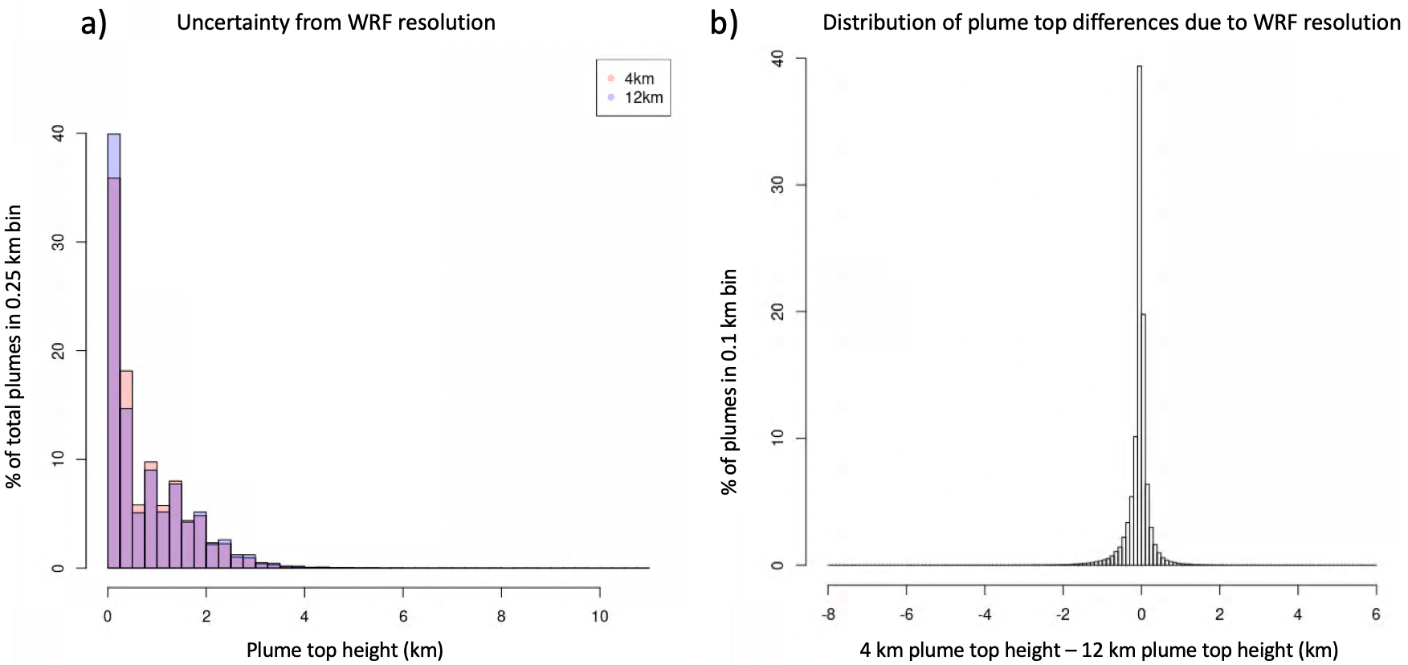 |
| --- |
| **Figure S4. (a)** A comparison of the distributions of plume top heights for 2018 plumes falling within WRF domain d02 (see fig. S3, n = 182,755) when using 4 km meteorological inputs (pink) and 12 km meteorological inputs (blue). Values are binned at 250 m intervals. **(b)** A histogram of the distribution of differences (4 km – 12 km) in the simulated wildfire plume top height when using 4 km meteorology versus 12 km meteorology for input to the F2010 model. Data reflects all 2018 plumes falling within WRF domain d02 and is binned at 100 m intervals. |

**S4. Linking wildfire heat fluxes and wildfire burned area**

Wildfire heat fluxes derived from QFED emissions and wildfire burned area polygons (MCD64A1 dataset) were linked using the following 3 approaches:

1. Closest in time: Closest in time linkages were determined by looking for the burned area polygons that presented the closest in time (days) intersections with grid cells possessing non-zero wildfire heat fluxes. Burned area polygons were checked for intersections with the gridded heat flux beginning with burned area polygons flagged as burning on the same day as the heat flux and iterating out to +/- 8 days. All polygons intersecting the non-zero heat flux on the closest in time date were then considered to play a role in generating that heat flux (fig. S5a). In cases where a burned area polygon is assigned as a closest in time polygon for heat fluxes on consecutive days, the polygon area is divided between the two days, with proportions following the ratio of their heat flux values.
2. Only option: Only option linkages were determined as cases where a burned area polygon intersects only a single gridded heat flux within the +/- 8 day window but falls beyond the closest in time threshold (fig. S5a). Effectively, this burned area could only reasonably be allocated to a single heat flux, suggesting that instrument temporal uncertainty may explain prior exclusion from the closest in time polygons.
3. Directional Spread: Directional spread linkages were made in reference to unallocated burned area polygons intersecting a non-zero heat flux within the +/- 8 day window that did not meet the criteria for closest in time polygons or only option polygons, but were in direct contact with burned area polygons that met these criteria. If the unassigned polygon was in contact with other burned area polygons that all burned on the same day or consecutive days, it was assigned to the heat flux on that day or divided into 2-pieces (weighted by relative heat fluxes) and allocated to the heat fluxes on each of the consecutive days (fig. S5b). We are operating under the assumption that this polygon was not previously assigned to the heat flux as a result of burn date uncertainty and employ the idea of a directional fire spread to allocate the burned area.

| 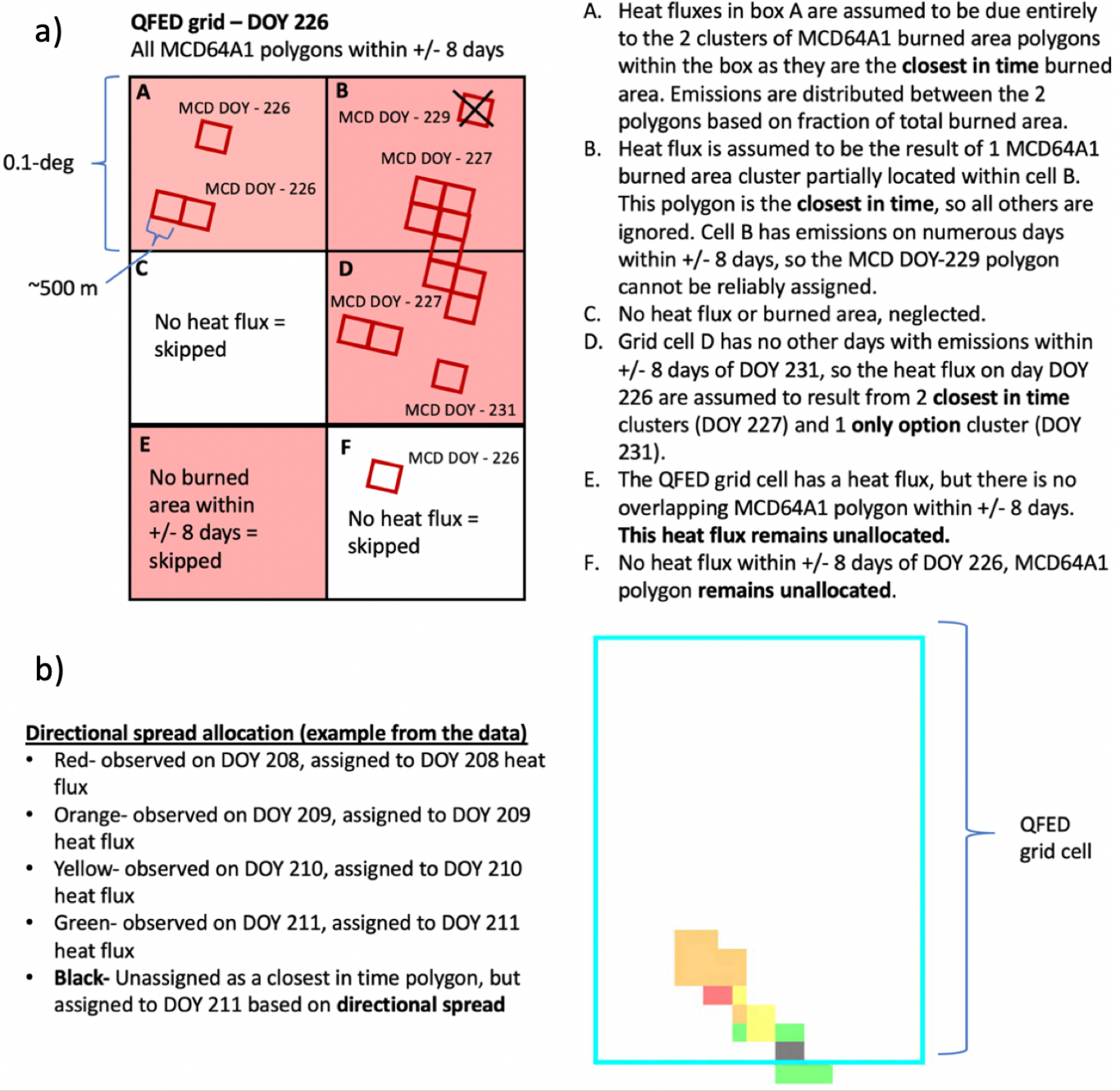 |
| --- |
| **Figure S5. (a)** A visual example of the burned are polygons overlaid on gridded heat flux data, with cases of closest in time burned area allocations, only option burned area allocations, and unallocated burned area and heat fluxes. Red grid cells represent the presence of a QFED derived heat flux, while white grid cells possess no QFED derived heat flux. **(b)** A visual representation of directional spread burned area polygon assignment pulled directly from the data. The black burned area polygon is assigned based on the notion of directional fire spread between the two green polygons. |

| Year | % burned area allocated - total | % burned area allocated - possible | % heat fluxes allocated - total |
| --- | --- | --- | --- |
| 2003 | 71.02 | 89.29 | 88.99 |
| 2004 | 63.99 | 86.98 | 83.26 |
| 2005 | 41.17 | 80.98 | 74.79 |
| 2006 | 76.81 | 92.31 | 91.08 |
| 2007 | 55.89 | 79.31 | 91.94 |
| 2008 | 64.00 | 83.02 | 85.16 |
| 2009 | 77.99 | 93.91 | 85.90 |
| 2010 | 66.96 | 83.75 | 88.45 |
| 2011 | 64.98 | 94.41 | 77.30 |
| 2012 | 76.24 | 90.58 | 95.21 |
| 2013 | 78.46 | 90.46 | 91.04 |
| 2014 | 57.18 | 78.90 | 89.02 |
| 2015 | 84.46 | 94.17 | 91.90 |
| 2016 | 72.65 | 91.59 | 85.93 |
| 2017 | 76.03 | 90.89 | 95.57 |
| 2018 | 84.57 | 93.94 | 94.47 |
| 2019 | 45.75 | 72.70 | 70.58 |
| 2020 | 81.39 | 91.60 | 96.81 |
| **Table S2.** A table of the annual percentages of allocated wildfire burned area and wildfire heat fluxes based on the methods used for generating inputs to the F2010 model. The distinction between total burned area and possible burned area allocation is made on the grounds that some MCD64A1 burned area polygons present no overlap with QFED derived heat fluxes whatsoever. A likely explanation for this lack of overlap stems from the temporal uncertainty of burned area observations. Generally, years characterized by greater wildfire activity are similarly characterized by greater percentages of allocation. This relationship is likely the result of resolution limitations to observation, such that more intense wildfire activity is more readily observed. | | | |
